# Atomic Undercoordination in Ag Islands on Ru(0001) Grown via Size-Selected Cluster Deposition: An Experimental and Theoretical High-Resolution Core-Level Photoemission Study

**DOI:** 10.1021/acs.jpcc.1c02327

**Published:** 2021-04-28

**Authors:** Luca Sbuelz, Federico Loi, Monica Pozzo, Luca Bignardi, Eugenio Nicolini, Paolo Lacovig, Ezequiel Tosi, Silvano Lizzit, Aras Kartouzian, Ueli Heiz, Dario Alfé, Alessandro Baraldi

**Affiliations:** † Department of Physics, University of Trieste, Via Valerio 2, 34127 Trieste, Italy; ‡ Department of Earth Sciences and London Centre for Nanotechnology, University College London, Gower Street, London WC1E 6BT, U.K.; § Elettra-Sincrotrone Trieste, S. S. 14, km 163.5 in AREA Science Park, 34149 Trieste, Italy; ∥ Department of Chemistry, Technical University of Munich, Lichenbergstrasse 4, 85748 Garching, Germany; ⊥ Dipartimento di Fisica Ettore Pancini, Universitá di Napoli Federico II, Monte S. Angelo, I-80126 Napoli, Italy

## Abstract

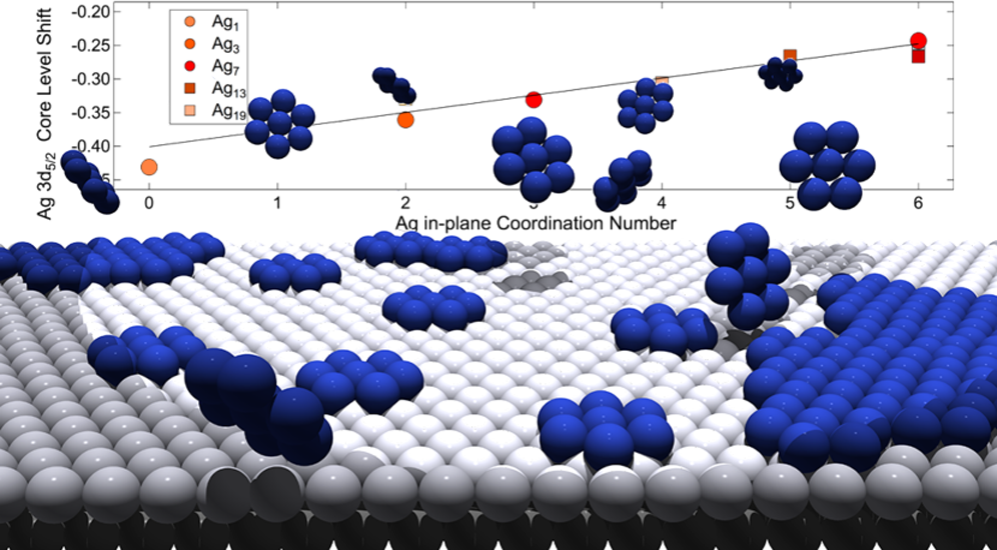

The possibility of depositing precisely mass-selected Ag clusters (Ag_1_, Ag_3_, and Ag_7_) on Ru(0001) was instrumental in determining the importance of the in-plane coordination number (CN) and allowed us to establish a linear dependence of the Ag 3d_5/2_ core-level shift on CN. The fast cluster surface diffusion at room temperature, caused by the low interaction between silver and ruthenium, leads to the formation of islands with a low degree of ordering, as evidenced by the high density of low-coordinated atomic configurations, in particular CN = 4 and 5. On the contrary, islands formed upon Ag_7_ deposition show a higher density of atoms with CN = 6, thus indicating the formation of islands with a close-packed atomic arrangement. This combined experimental and theoretical approach, when applied to clusters of different elements, offers the perspective to reveal nonequivalent local configurations in two-dimensional (2D) materials grown using different building blocks, with potential implications in understanding electronic and reactivity properties at the atomic level.

## Introduction

The atomic coordination number (CN) plays a crucial role in determining physical and chemical properties in condensed matter. The importance of this quantity is well recognized in the field of heterogeneous catalysis.^1^ It is well-established that the chemical properties for several systems are determined by the presence of a large population of atoms sitting at defect sites, edges, corners, and nanofacets in catalytic active nanoparticles.^2,3^ The importance of these local configurations was revealed both in noble^4,5^ and transition metal^6,7^ nanoparticles, for which it has been demonstrated that a high concentration of low-coordination atomic sites results in increased chemical reactivity. An example of this phenomenon is the promotion of the O_2_ molecular adsorption and dissociation,^4^ the hydrocarbon dehydrogenation,^8^ and the chemical conversion of *N*-heterocyclic carbene molecules.^9^ Besides nanoparticles, the presence of these specific configurations is known to contribute to the chemical reactivity of solid surfaces as well. According to the Hammer–Nørskov model,^10,11^ the main reason for the high adsorption energies and for the energy barrier reduction for molecular dissociation on transition metal surfaces can be explained in terms of the d-band center shift on undercoordinated atoms. This is true also in the case of the bimetallic surfaces and interfaces and for near-surface alloy catalysts.^12^ For these systems, the different structural and morphological properties of the surface, which determine the presence of undercoordinated atoms, are driven by the interplay between atom–atom and atom–substrate interactions. The same quantities are used in conjunction with diffusion properties, to determine growth, nucleation, and aggregation processes,^13^ which in turn define the presence of undercoordinated atomic species in the adlayer structure.

The possibility of discriminating nonequivalent atoms of the same species sitting on solid surfaces in different local environments, and in particular with different CNs, is a well-known characteristic of high-resolution core-level spectroscopy (HR-CLS).^14^ This experimental approach has been employed to distinguish top-layer atoms that have a number of broken bonds with respect to the bulk ones,^15−18^ atoms at step edges,^19^ strained stepped surfaces,^20^ adatoms/ad-dimers in homo-^21,22^ and heteroatomic surfaces,^23^ for atomic nanowires,^24^ edge atoms in two-dimensional (2D) materials,^25^ and for nanoclusters grown on graphene.^26^ In the case of single-layer metal islands supported on different metallic substrates, the ability to distinguish atoms with different CNs depends on the surface density of these local configurations, which in turn depends on the extension of the islands and the degree of surface order. In fact, the greater the latter, the lower the density of the undercoordinated atoms sitting on the periphery of the islands or which are incorporated into disordered islands, where the in-plane coordination is not equal to an ideal close-packed configuration.

When species A is deposited, for example, on an fcc(111) or hcp(0001) surface of metal B, the possible in-plane CN ranges from CN_A_ = 0 (single monomer) to CN_A_ = 6 (complete monolayer), while CN_B_, which is the coordination number with the substrate, is CN_B_ = 3 for threefold adsorption sites and CN_B_ = 1 for atop sites (for both monomers and atoms of a complete single layer). Atomic undercoordination modifies the core level from its value of an isolated atom because of the presence of neighboring atoms (tight-binding theory); on the other hand, the atoms are fully coordinated when we consider atoms belonging to the second and lower layers at solid–vacuum interfaces.

In this experimental and theoretical investigation, we employ HR-CLS to study the coordination effects on single-layer Ag islands prepared by depositing size-selected clusters of Ag_1_ (monomers), Ag_3_ (trimers), and Ag_7_ (heptamers) on Ru(0001). Clusters of this size, used as building blocks for the formation of two-dimensional islands, allow us to exploit their different individual structures to create configurations with a different density of undercoordinated atoms in a two-dimensional Ag layer. However, silver has a peculiar valence band electronic structure, which makes the distinction of the effects of reduced surface coordination arduous to reveal using HR-CLS.^15^ In addition to that, the detection of the local atomic configuration with a small CN is complicated by the process of cluster surface diffusion. Therefore, one expects that when Ag clusters are deposited on Ru(0001), they will diffuse and join other clusters to form small islands, which then can act as centers of nucleation for larger structures or can further diffuse on the surface. The latter process will depend on substrate temperature and island critical size for the diffusion process which, thanks to the immiscibility of Ag in Ru,^27^ takes place likely with simple hopping events among nearest neighbor sites.

## Methods

### Experimental Methods

The measurements were performed in an ultrahigh vacuum (UHV) chamber at the SuperESCA beamline of the Elettra synchrotron radiation facility in Trieste, Italy. The photoemission spectra were collected by means of a Phoibos 150 mm mean radius hemispherical electron energy analyzer from SPECS, equipped with an in-house developed delay line detector. The overall experimental energy resolution (which accounts for both the electron energy analyzer and the X-ray monochromator) was always kept within 70 meV in all measurements, as determined by probing the width of the Fermi level of an Ag polycrystal.

The Ru(0001) single crystal was mounted on a sample manipulator with 4 degrees of freedom. The sample was heated by electron bombardment from hot tungsten filaments mounted behind it. The temperature of the sample was monitored by means of two K–type thermocouples directly spot-welded on the back of the specimen. The Ru(0001) surface was cleaned by repeated cycles of Ar^+^ sputtering and flash annealing to 1500 K, followed by annealing in O_2_ and in H_2_ gas.^28,29^ The surface long-range order and cleanliness were checked by low-energy electron diffraction, which exhibited a sharp (1 × 1) pattern with low background intensity, and by X-ray photoelectron spectroscopy (XPS). During cluster deposition and photoemission measurements, the background pressure was always kept below 1.5 × 10^–10^ mbar, with just the He pressure rising up to 10^–8^ mbar, as monitored with a mass spectrometer.

The Ag clusters were deposited on Ru(0001) at room temperature using ENAC (Exact Number of Atoms in each Cluster), a size-selected nanocluster source based on laser ablation/supersonic aggregation, which was developed and built at the Nanoscale Materials Laboratory of Elettra Sincrotrone Trieste. The working principles on which ENAC relies follow the design of a similar size-selected cluster source developed at the Technical University of Munich,^30^ which has been successfully used in the past years to perform experiments involving Ag clusters.^31−34^ ENAC has been developed to produce size-selected nanoclusters in UHV conditions and its design was optimized to connect it to the experimental station of the SuperESCA beamline, to perform in situ characterization of the clusters using synchrotron radiation. ENAC is composed of five vacuum chambers equipped with a differential pumping system and a set of ion optics to transport the nanoclusters and perform the mass selection. In the first stage, ablation of a metal target takes place using a pulsed (120 Hz) Nd-YAG laser operating at 532 nm. The target is constantly kept in motion to avoid its perforation. The ablation process produces a metallic plasma that is transported by a He gas pulse into a thermalization chamber, where it undergoes a supersonic adiabatic expansion. In the second and third stages, the charged nanoclusters are guided through a 500 mm home-built radiofrequency octupole set to transport nanoclusters with masses from 10 to 16 000 amu. The positively charged nanoclusters are then separated from negatively charged and neutral ones using an electrostatic quadrupole bender. The size selection occurs in the fourth stage of the cluster source using a quadrupole mass spectrometer (QMS) that can provide high mass resolution up to a single amu in a wide range from 1 to 16 000 amu. After the QMS, the size-selected nanoclusters are further transported through the fifth stage using a second radiofrequency octupole and then focused with an Einzel lens on the sample located in the SuperESCA preparation UHV chamber. The number of clusters reaching the Ru(0001) surface can be monitored by reading the current on the sample itself. Soft landing conditions^35^ were achieved by controlling the kinetic energy of the ionized cluster, which was kept below 1 eV/atom. A mass spectrum performed with ENAC is presented in the Supporting Information for Ag clusters made of a number of atoms from 1 to 46.

The XPS spectra were acquired by tuning the photon energy to have a photoelectron kinetic energy of about 100 eV to enhance surface sensitivity. For each spectrum, the photoemission intensity was normalized to the photon flux and the binding energy (BE) scale was aligned to the Fermi level of the Ru substrate. Doniach–Šunjić line profiles have been used to fit each spectral component, convoluted with a Gaussian distribution, which accounts for the experimental, phonon, and inhomogeneous broadening.^36^

### Theoretical Methods

The calculations have been performed using density functional theory (DFT) as implemented in the VASP code.^37^ The atomic structure of the studied systems was fully relaxed until the largest residual force was less than 0.02 eV/Å. We employed the projector augmented wave (PAW) method^38^ to account for the core electrons, using PBE potentials,^39^ with 8 and 11 electrons in the valence band for Ru and Ag, respectively. Single-particle orbitals were expanded in plane waves using a kinetic energy cutoff of 400 eV and the relaxations were performed using a 3 × 3 × 1 uniform grid to k-points to sample the Brillouin zone.

The Ru(0001) surface was modeled with a slab geometry, using a (5 × 5) supercell with four layers, of which the bottom two were kept frozen at the bulk interatomic distances. The vacuum distance between the two surfaces of the periodically repeated slabs was at least 13 Å for the clean and Ag cluster-covered surface. Core-level binding energies have been estimated within the final state approximation. Diffusion barriers were estimated with the climbing-image Nudged Elastic Band method.^40^

## Results and Discussion

The first step of the experiment was the measurement of the 3d_5/2_ core-level spectrum of a Ag(111) single crystal, to establish the most accurate way to measure the core electron binding energy (BE) of bulk atoms. This served as a reference for comparing the experimental and theoretical results and will be used to convert theoretically calculated BE shifts into absolute BE values. As anticipated, the measurement of the surface core-level shift (SCLS is the shift between the core level of the surface atoms with respect to the bulk ones) in silver is a very challenging task. Andersen et al.^15^ have found that the surface component of the 3d_5/2_ spectrum of clean Ag(111) is indeed difficult to discern from the bulk component, presenting an SCLS that was estimated to be ca. 100 meV toward lower BEs. Such a small value is caused by final state effects, namely, by the core–hole screening properties of the 5s valence band electrons and by the reduced electron density of states at the Fermi energy of noble metals with respect to transition metals, where SCLSs are typically much larger.

**Figure 1:**
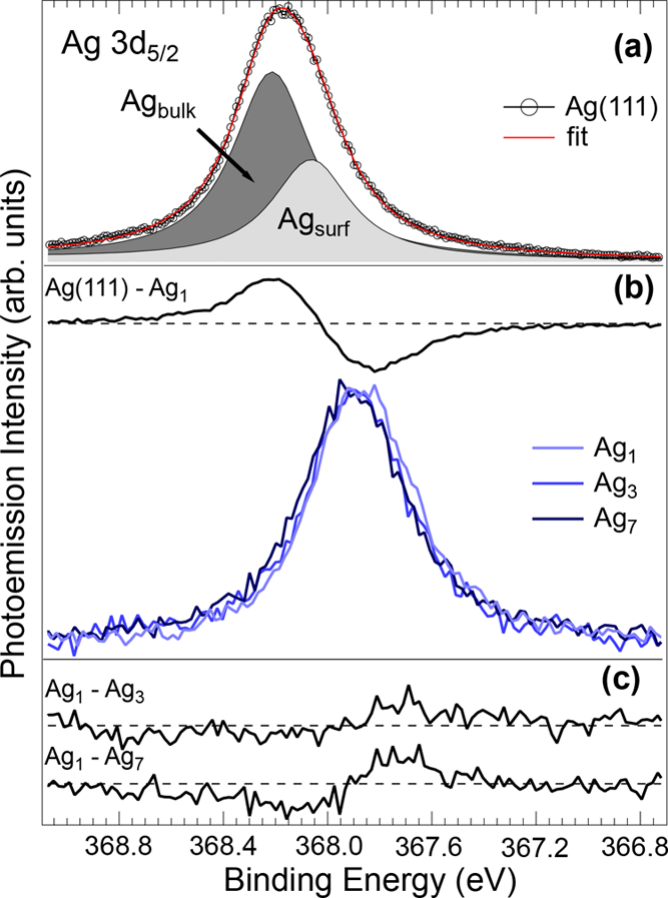
(a) Ag 3d_5/2_ core-level spectra from the Ag(111) surface recorded at a photon energy of 470 eV and *T* = 300 K. The spectral components obtained by the fit originate from bulk (Ag_bulk_) and first-layer (Ag_surf_) atoms. (b) Ag 3d_5/2_ core-level spectra measured after deposition of Ag_1_ (0.0051 ML), Ag_3_ (0.0067 ML), and Ag_7_ (0.0073 ML) clusters on Ru(0001) and the difference spectrum Ag(111)–Ag_1_, divided by 4. (c) Ag 3d_5/2_ difference spectra Ag_1_–Ag_3_ and Ag_1_–Ag_7_.

Figure 1a shows the Ag 3d_5/2_ spectrum of the clean Ag(111) surface measured with a photon energy of 470 eV, together with the spectral decomposition. The unusual asymmetric spectral lineshape toward lower BEs, which cannot be justified by electron–hole pair excitation or energy losses, as it would result in the build-up of a tail at higher BEs, clearly reveals the presence of a multicomponent structure The spectrum can be properly fitted with two components at 368.20 and 368.05 eV, corresponding to bulk (Ag_bulk_) and first-layer (Ag_surface_) components. The values for the lineshape parameters Γ (Lorentzian), *G* (Gaussian), and α (asymmetry) resulting from the fit were 340 meV, 70 meV, and 0 for both bulk and surface components, respectively. The latter value confirms a low density of states at the Fermi level, as expected for a noble metal. This peak assignment is based on the fact that the 3d_5/2_ spectrum measured at high photon energy (650 eV) shows a reduced spectral weight at lower binding energies. This can be interpreted based on the increased mean free path of photoelectrons at higher kinetic energies. This assignment is confirmed by DFT calculations of the SCLS, which was estimated to be of −146 ± 20 meV with respect to the bulk component, in very good agreement with our experimental outcomes.

The high-resolution Ag 3d_5/2_ spectra measured upon deposition of Ag_1_, Ag_3_, and Ag_7_ clusters on the Ru(0001) surface are reported in Figure 1b together with the difference spectrum Ag(111)–Ag_1_, divided by 4. The total Ag coverages for the three depositions were 5.1 × 10^–3^ ML (Ag_1_), 6.7 × 10^–3^ ML (Ag_3_), and 7.3 × 10^–3^ ML (Ag_7_), where 1 ML (monolayer) is equal to 1.38 × 10^15^ atoms/cm^2^. Such very small surface coverages were dictated by the idea of preventing cluster collisions, merging already upon deposition, and hindering the island nucleation process. The main results from this experiment were (i) an overall core-level shift toward lower BEs with respect to the bulk Ag_bulk_ component, which amounts to about −400 meV and (ii) a shift between the spectra that inversely decreases with the size of the deposited clusters. Even if the spectral differences are not large, it is possible to appreciate the shift of the spectral maximum, given the good signal-to-noise ratio (considering the extremely small Ag coverage). The difference spectra, plotted in Figure 1c, show the shift of the spectral intensity toward higher BEs as the cluster sizes increase.

**Figure 2:**
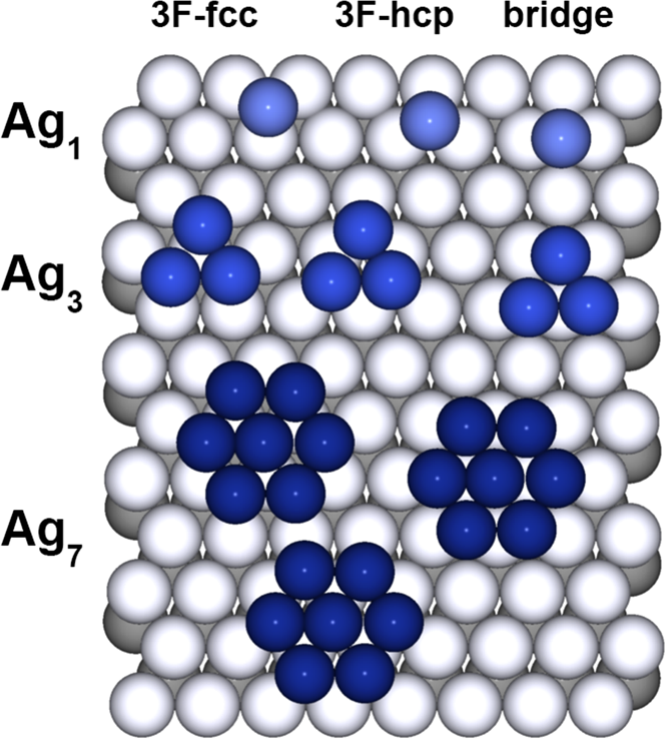
Different adsorption configurations of Ag_1_, Ag_3_, and Ag_7_ clusters tested in the DFT calculations.

To obtain a thorough understanding of the experimentally prepared system, we resorted to DFT calculations, initially investigating the structure and preferred adsorption sites for the three different types of clusters. It should be emphasized that the monodisperse clusters produced by ENAC and guided to the surface sample are positively charged. For these reasons, we considered the starting cluster geometry to be the minimum energy configuration structure that Ag_1_^+^, Ag_3_^+^, and Ag_7_^+^ adopt in the gas phase (GP). In agreement with previous theoretical calculations,^41^ we found that the preferred configuration for Ag_3_^+^ is an equilateral triangle, while for Ag_7_^+^, it corresponds to a planar hexagonal wheel with an atom in the center. The configurations studied on the Ru(0001) are shown in Figure 2. The deposition of the clusters is performed in soft landing conditions, thus strongly reducing the possible breakup of the Ag–Ag bonds that the cluster could experience upon impacting the surface.

We tested the energetics for adsorption in 3-fold fcc, 3-fold hcp, bridge, and on-top sites. The test on the latter site has been performed only for Ag_1_ since it appeared to be markedly the least favorite configuration. Monomers, trimers, and heptamers in fcc and hcp sites resulted in equally stable adsorption configurations (within the numerical errors) and are only slightly preferred (by about 50 meV) than the bridge configuration (see Table 1). The results, which for single adatoms turn out to be in very good agreement with previous DFT calculations,^42^ show that the adsorption energy/atoms ratio increases with the local density of Ag atoms, i.e., by spanning from monomers to heptamers. This fact indicates an attractive Ag–Ag interaction and can explain the tendency of Ag to form on Ru(0001) small compact islands at low coverage and temperature.

**Table 1:** Adsorption Energies (in eV) for the Different Combinations of Clusters and Adsorption Sites Investigated in the DFT Calcualtions

cluster	fcc	hcp	bridge	top
Ag_1_	2.483	2.483	2.424	2.076
Ag_3_	2.57	2.566	2.519	
Ag_7_	2.647	2.646	2.602	

Similar adsorption energies for the two nonequivalent 3-fold adsorption sites are due to the very large covalent radius of Ag atoms (1.53 Å) and indicate therefore a negligible influence of the second layer Ru atoms.

**Figure 3:**
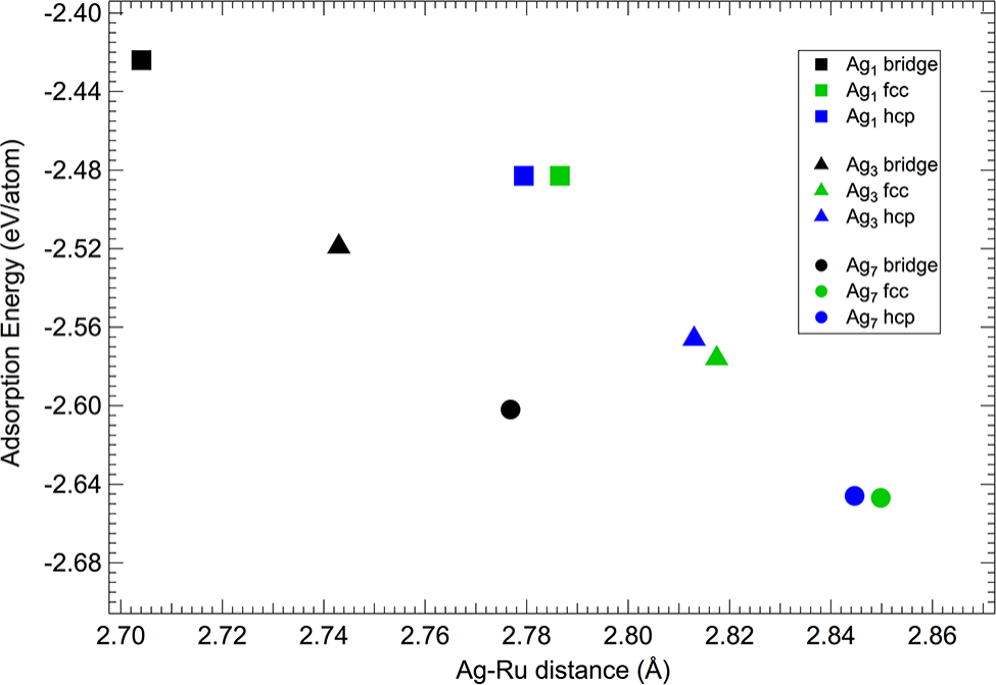
Adsorption energy per Ag atom as a function of average Ag–Ru distance for Ag_1_, Ag_3_, and Ag_7_ clusters on Ru(0001).

The role of the Ru substrate is reduced when increasing the cluster size and the local density of Ag atoms. This is also confirmed by the heights of Ag atoms with respect to the Ru first layer, which for the most stable adsorption configurations (fcc and hcp sites) goes from 2.78 Å for Ag_1_ (a value that is in excellent agreement with previous findings^42^) to 2.84 Å (Ag_7_), as shown in Figure 3. This suggests that the Ag–Ag interatomic interactions become larger when increasing the Ag island size.

**Figure 4:**
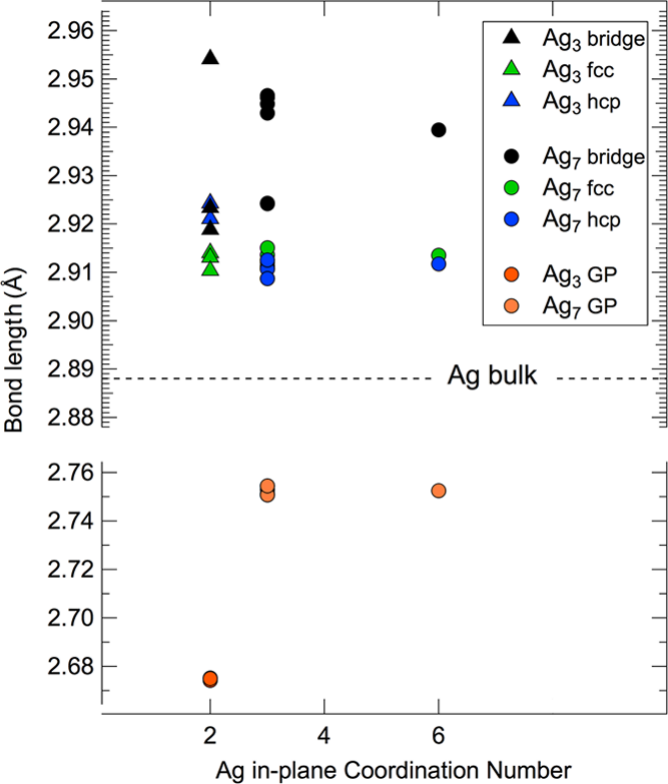
Calculated bond length for Ag_3_ and Ag_7_ clusters both in the gas phase (GP) and for different adsorption configurations on the Ru(0001) surface. The value for Ag in the bulk is reported for reference.

A further significant aspect of this analysis is the understanding of the dependence of the Ag–Ag bond length on the number of atoms in the islands, thus addressing the issue of induced strain in the Ag bonds.^43^ In figure 4, we report the calculated bond length for Ag clusters composed of different numbers of atoms and adsorbed in different sites on the Ru(0001) surface. The distance reported in the figure was obtained by calculating the average distance from the nearest neighbors for each atom in the clusters. We report as well the values calculated for Ag_3_ and Ag_7_ in the gas phase (GP) as a reference. We can observe that there is no significant dependence of the bond length on the in-plane CN. In the gas phase, we observe a shrinking of ca. 7% of the bond length for the Ag_3_ cluster, while the contraction is smaller for Ag_7_ (ca. 4%). On the other hand, we observe an expansion of the bond length when the clusters are adsorbed on the surface, with a dependence on both the size of the cluster and the adsorption site. This strain ranges from ca. 3% for Ag_7_ on bridge adsorption sites to about 1% for Ag_7_ on 3-fold adsorption sites, with adsorption on the fcc and hcp configurations being almost equivalent.

**Figure 5:**
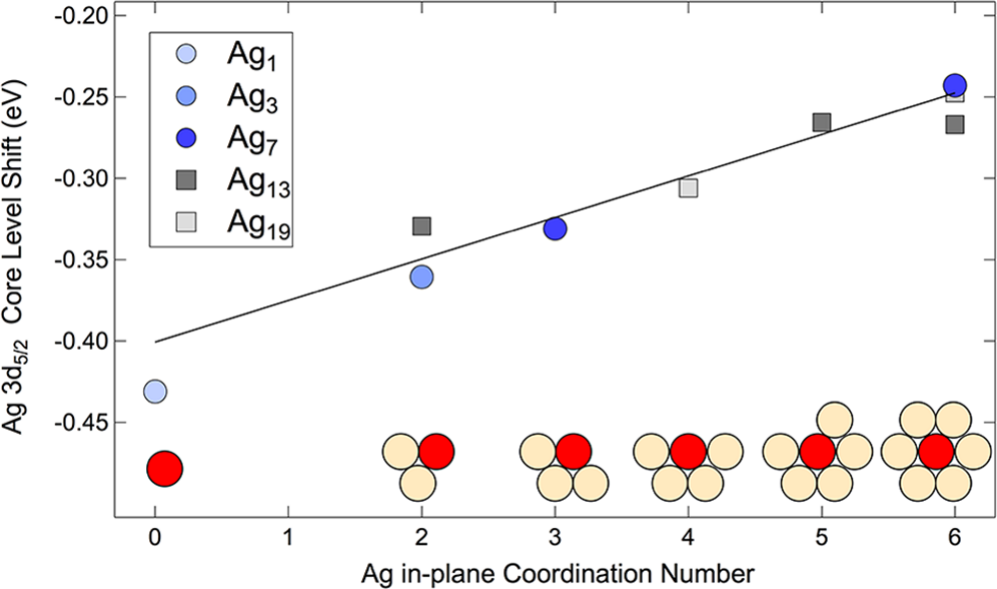
Calculated Ag 3d_5/2_ core electron binding energy shifts dependence on the in-plane coordination number (CN) for different local configurations. The values are reported with respect to the binding energy of bulk atoms.

The difference induced by the in-plane CN can be appreciated by observing the trend of the calculated 3d_5/2_ core-level shifts in the various Ag structures with different coordination numbers, as reported in Figure 5. To increase the data set and to probe the changes in 3d_5/2_ core electron binding energies caused by different CNs, we also investigated the effects caused by increasing island sizes, simulating structures with Ag atoms in a hexagonal close-packed arrangement, as for the Ag_7_ case, but forming larger clusters/islands. We expect that all types of clusters, in particular monomers because of a diffusion energy of only 59 meV we found using NEB (in agreement with the value reported in ref (42)), will indeed diffuse on the Ru(0001) surface and start forming islands with different sizes. If this would be the case, the density of undercoordinated atoms would be lower than in the situation with clusters randomly distributed on the surface. The two configurations used to model this conjecture (shown in the Supporting Information) are composed of Ag atoms with CN ranging from 6 to 2. Ag dimers (CN = 1) have not been considered in this calculation and thus are absent in Figure 5.

Figure 5 shows a remarkable, and up to now never detected, linear dependence of the core-level shift in a bimetallic surface composition with the overall in-plane coordination, with a proportionality coefficient between the shift and the number of Ag nearest neighbors of 25 ± 5 meV per bond. The very small shift predicted by the theoretical calculations further proves that measuring the structure-related properties of undercoordinated Ag clusters/islands on a transition metal surface is extremely challenging. When we compare the changes in core levels of undercoordinated configurations (but in this case, for three-dimensional (3D) systems) obtained upon formation of Rh adatoms and ad-dimers on (111) and (100) rhodium surfaces,^21^ a value of 130 meV per bond (3d_5/2_ core level) is larger by a factor of 5 with respect the one obtained for our system. A large difference has also been obtained in the case of undercoordinated Pt atoms when measuring the Pt 4f_7/2_ core-level shift for which a value of 120 meV per bond was found.^22^

**Figure 6:**
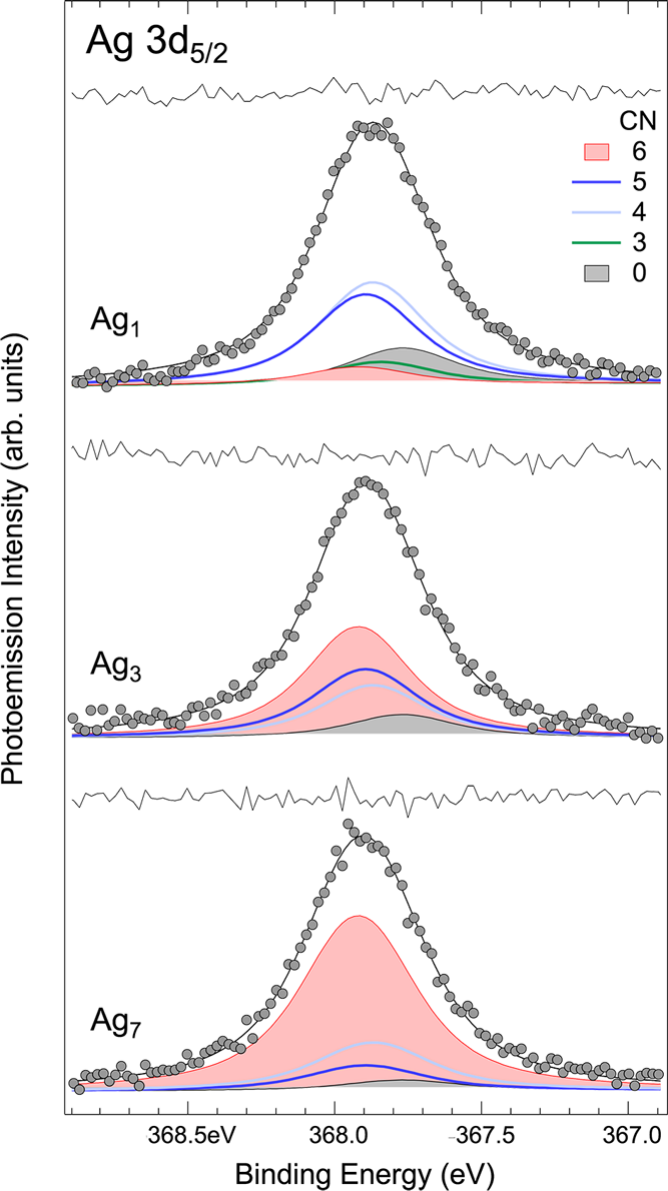
Ag 3d_5/2_ core-level spectra from Ag_1_, Ag_3_, and Ag_7_ clusters deposited on Ru(0001). Spectra are collected using a photon energy of 470 eV. Each spectrum is fitted using seven components separated by 25 meV, according to the DFT results.

The calculated CN-dependent core electron binding energy shift was used to fit the Ag 3d_5/2_ spectra measured after deposition of Ag_1_, Ag_3_, and Ag_7_ on Ru(0001), as shown in Figure 6, using a method already applied in the case of the analysis of the C 1s spectrum of a graphene layer on Re(0001)^44^ and Ru(0001).^45^ Each spectrum has been fitted using seven components spaced by 25 ± 5 meV, each corresponding to a different CN ranging from CN = 0 (monomer) to CN = 6 (Ag monolayer). It was possible to obtain the absolute BE of each component combining the experimental and theoretical work performed considering a Ag(111) single crystal. The relative CL shifts were calculated using as a reference the theoretical BE of atoms in the bulk of a Ag(111) single crystal previously simulated; subsequently, this value has been aligned to the experimentally measured one, as extrapolated from the Ag 3d_5/2_ core-level spectrum reported in Figure 1a. Given this assumption, the core electron binding energy shifts can be considered as the experimental values, and the absolute BE of the components associated to each CN can easily be obtained by applying a shift to this value. The Γ and α parameters were kept fixed to the bulk values, while *G* was calculated to be equal to 0.20, 0.19, and 0.23 eV, respectively, for the Ag_1_, Ag_3_, and Ag_7_ depositions.

The modification in the spectral weight previously discussed is confirmed by the data analysis, showing that after the deposition of the Ag_7_ clusters, most of the atoms are in CN = 6. This clearly suggests that Ag_7_ is very mobile even at *T* = 300 K and quite large islands are present on the surface after deposition. Since the populations of CN = 5 and 4 atoms are very low, the islands formed are expected to have a high degree of order, thus suggesting that Ag_7_ is a good building block to form a hexagonal close-packed atomic arrangement. Consequently, if we ideally think of Ag_7_ as flat and unbreakable clusters, it is possible to form a 2D Ag layer by simply aligning the clusters along the <120> direction, forming monolayer islands having atoms in the inner part with CN = 6 and atoms at the edges with CN = 4 and 3. While the population of 5 and 4 species is very small, the component related to CN = 3 cannot be detected within our error bar. The good mobility of Ag_7_, which is known to be related to decreased cluster mobility (see for example Re clusters on Ru(0001)^46^), could be related to the large lattice mismatch between the Ag_7_ cluster and Ru, with the average distance between the atoms of the first (2.91 Å) being 7.4% larger than the lattice parameter of Ru (2.71 Å), which is known to reduce the overall Ag–Ru affinity and hence increase the Ag cluster mobility. A further indication of the weak interaction of Ag_7_ with the substrate is that such configuration is characterized by the highest height of Ag atoms with respect to the Ru first layer, as previously discussed in Figure 3. The presence of a small component related to Ag_1_ indicates that a limited part of Ag_7_ clusters dissociates upon impacting the surface forming monomers, which remain on the surface as isolated atoms. Even if the cluster energy increases with size, we cannot exclude the activation of an Ostwald ripening process where the Ag atoms at the edges of the clusters detach from Ag_7_ and migrate on the surface. This is indeed the process found in the case of monodisperse Pd_19_ clusters deposited on Rh(111).^47^ However, contrary to our system, the Pd–Pd binding energy is considerably lower than the binding energy of the cluster to the substrate. The spectrum measured after deposition of Ag_3_ clusters shows a reduced population of atoms with CN = 6 with a corresponding increase of atoms with few in-plane nearest neighbors, thus supporting the idea that even for these clusters, the formation of 2D Ag island with the high density of defects is dominant.

Surprisingly, the Ag 3d_5/2_ spectrum for the Ag_1_ deposition indicates that there is a very small number of atoms in a close-packed arrangement. To explain this unforeseen result, we can take into account that the deposition took place at a substrate temperature (300 K) much lower than the value used to achieve a well ordered Ag adlayer structure on Ru(0001), which is between 690^48^ and 790 K.^49^ Despite the fact that the deposition temperature we used was high enough to make the Ag atoms highly mobile on this surface, it could not be sufficient to promote the formation of a full close-packed atomic arrangement, producing instead a lot of local defects (CN = 5 and 4). For a similar system, i.e., Au on Ru(0001),^50^ the room temperature deposition resulted in the formation of a dendritic structure caused by kinetic limitations, which was found to be thermodynamically unstable.^51^ According to the Witten and Sander aggregation model,^52^ this effect can be due to the lower mobility of adatoms on the periphery of the noble metal islands. The presence of a disordered layer upon Ag_1_ deposition is also supported by the large density of Ag atoms with CN = 5, with this value being incompatible with the formation of high-symmetry and low-energy Ag islands with quasihexagonal symmetry, which are energetically favorite. In this case, besides the number of Ag atoms with CN = 6 in the inner part of the island, we would expect to have atoms with CN = 3 and 4 but not 5, independent of the island size (see the Supporting Information).

Besides the growth mode, we cannot ignore the role played by surface steps, which have been found to be the preferential attachment site in the case of the growth of Ag on Ru(0001) at room temperature.^53^ For a very similar system, although at much larger coverage with respect to the ones used in our work, i.e., deposition of 0.1 ML of Ag on Re(0001) at room temperature,^54^ atoms bind exclusively at step edges where they form irregularly shaped small atomic aggregates. In particular, it was found that adatoms do not wet step edges and are responsible for an irregular and fairly porous shape of individual Ag aggregates, with large “cavities” caused by a totally unidirectional growth. Since the electronic structure of Re is quite comparable to Ru, we can imagine a similar behavior in our systems, which would result in relevant populations of Ag atoms with CNs lower than 6.

Even if the motion of the island for several systems is related to the island size (diffusion coefficient scales as the inverse radius of the island^55^), especially in the case of Ag, there are examples in which the atomic island motion can take place also for extremely large islands. This can occur also by means of a collective diffusion mechanism involving each atom in the island, as in the case of relatively small homoepitaxial islands on fcc(111) transition metals. The atomistic mechanism could be different depending on the island size, ranging from a gliding process (*N* < 20), in which all of the atoms of the small island move simultaneously, to a kink dislocation motion (*N* > 100), for which an atomic row of atoms moves simultaneously from fcc to hcp three-fold sites.^56^

## Conclusions

In summary, by means of HR-CLS, we have investigated the local configurations of Ag atoms with a low CN obtained upon deposition of mass-selected Ag clusters on Ru(0001). We have found that the Ag 3d_5/2_ core-level shift shows a linear dependence on the in-plane CN. The very small core-level shift/bond and the large mobility of Ag clusters on the Ru surface are two intrinsic limitations, which hindered the possibility to distinguish clearly the spectral features originating by each nonequivalent atom in the cluster. On the other hand, our approach is potentially a starting point paving the way to the investigation of the properties of 2D materials grown using adsorbed clusters, which are expected to present, according to the different structures, a number of nonequivalent local configurations related to different CNs. We believe that this methodology could be the key to a deeper understanding of the factors driving the electronic and reactivity properties of undercoordinated atoms in 2D materials.

## Supporting Information Available

Mass spectrum of Ag^+^ clusters produced by the ENAC source and simulations with large Ag islands; mass spectrum obtained transporting and depositing on a Ru(0001) surface Ag cluster within the mass range of about 100–5000 amu; models used to perform the simulation with larger islands composed of (a) 13 and (b) 19 Ag atoms; and models and related histograms of the percentage of atoms for each CN in Ag islands with a hexagonal close-packed arrangement made of (a) 19, (b) 37, and (c) 61 Ag atoms (PDF)
