# Supplementary material for: Atomic Undercoordination in Ag Islands on Ru(0001) Grown via Size-Selected Cluster Deposition: An Experimental and Theoretical High-Resolution Core-Level Photoemission Study
Source: J Phys Chem C Nanomater Interfaces. 2021 Apr 28;125(17):9556–63. doi: 10.1021/acs.jpcc.1c02327 (PMC8279646; doi:10.1021/acs.jpcc.1c02327)
Supplement: Supplementary file 1 — jp1c02327_si_001.pdf [file jp1c02327_si_001.pdf]

Atomic Under-Coordination in Ag Islands on Ru(0001) Grown via Size-Selected Clusters Deposition: an Experimental and Theoretical High-Resolution Core Level Photoemission Study. Supporting Information.

Luca Sbuelz,[†] Federico Loi,[†] Monica Pozzo,[‡] Luca Bignardi,[†] Eugenio Nicolini,[¶] Paolo Lacovig,[¶] Ezequiel Tosi,[¶] Silvano Lizzit,[¶] Aras Kartouzian,[§] Ueli Heiz,[§] Dario Alfè,[‡,‖] and Alessandro Baraldi[*,†,¶]

†*Department of Physics, University of Trieste, via Valerio 2, 34127 Trieste, Italy.*

‡*Department of Earth Sciences and London Centre for Nanotechnology, University College London, Gower Street, London WC1E 6BT, UK.*

¶*Elettra-Sincrotrone Trieste, S. S. 14, km 163.5 in AREA Science Park, 34149 Trieste, Italy.*

§*Department of Chemistry, Technical University of Munich, Lichenbergstrasse 4, 85748*

‖*Dipartimento di Fisica Ettore Pancini, Università di Napoli Federico II, Monte S. Angelo, I-80126 Napoli, Italy.*

E-mail: baraldi@elettra.eu

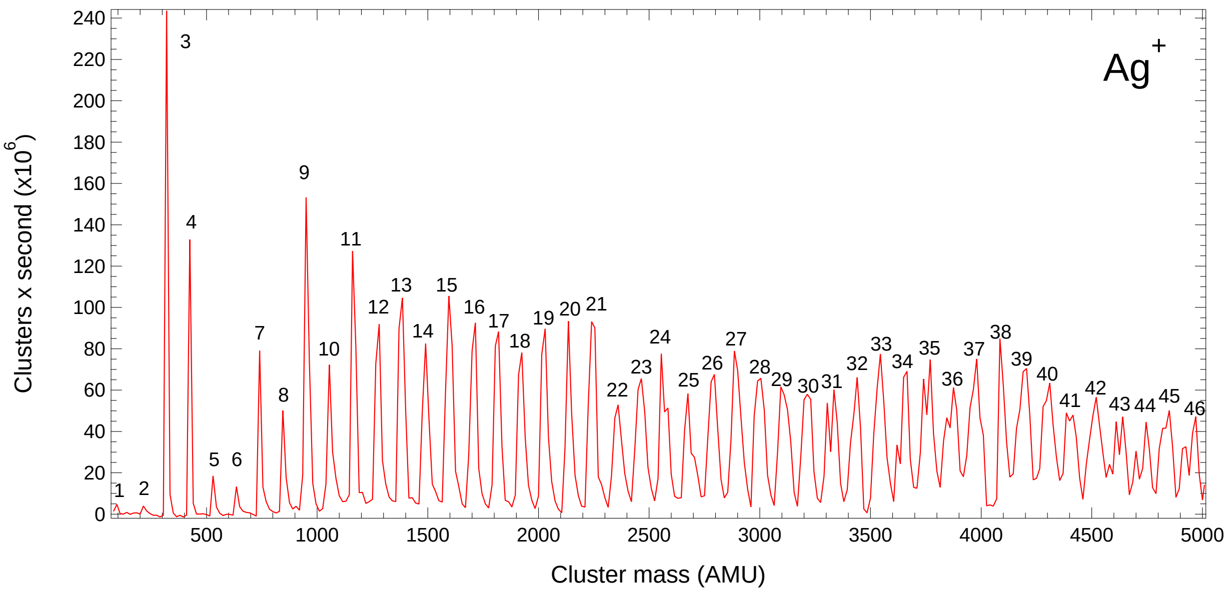

Figure S1: Mass spectrum obtained transporting and depositing on a Cu sample Ag cluster within the mass range of about 100-5000 a.m.u.

Mass spectrum of Ag^+ clusters

In Figure S1 we report a mass spectrum acquired using ENAC. The measurement was acquired gradually increasing the size of transported clusters thanks to the QMS from 100 to above 5000 a.m.u. and measuring the current reaching the sample.

Simulations with larger Ag islands

In Figure S2 we report the configurations used to study larger Ag islands made of 13 and 19 atoms. This work was performed in order to obtain a larger data set for the study of the changes in electron binding energies caused by different CN and to probe the effects caused by increasing the island size. The first island is built by adding one atom at the center of each side of the Ag_7 cluster, obtaining atoms with CN $=$ 2, 5 and 6. The second island is obtained by adding another atom at each corner of the initial hexagon. Considering the boundary conditions, this results in an island with external atoms with CN $=$ 4, and inner atoms with CN $=$ 6.

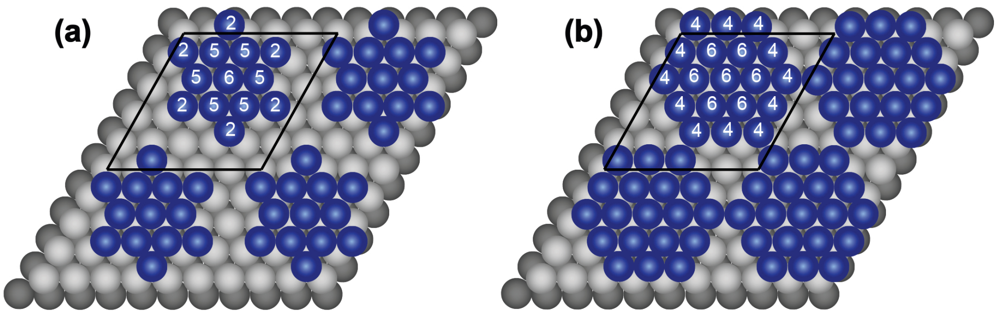

Figure S2: Models used to perform the simulation with larger islands composed of (a) 13 and (b) 19 atoms. The CN of each atom is reported on the figure and the unit cell is shown (black line).

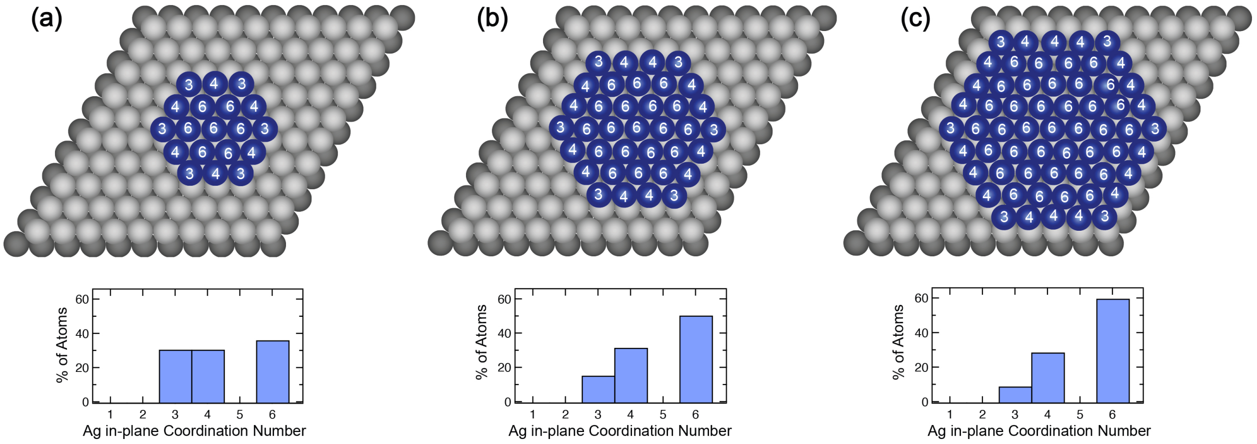

Figure S3: Models and related histograms of the percentage of atoms for each CN in Ag island with hexagonal closed-packed arrangement made of (a) 19, (b) 37 and (c) 61 atoms.

In Figure S3 we report three different systems with Ag island made of an increasing number of atoms: 19, 37 and 61. The islands are obtained by adding atoms until a full, ordered hexagonal closed-packed arrangement is obtained. The histograms attached to each system highlight how increasing the size of the islands modifies the amount of atoms with each CN: each system only has atoms with CN = 3, 4 and 6, but larger islands have an higher percentage of atoms with CN = 6. The fact that atoms with CN = 5 cannot exist in an island with an ordered hexagonal closed-packed arrangement means that atoms with such CN can be used as indicators of disorder. This is consistent with the conclusions of our work where we claim to obtain the highest degree of order depositing Ag_7, where we have the lowest population of atoms with CN = 5 among the three different depositions.
